# Trust and easy access to home care staff are associated with older adults’ sense of security: a Swedish longitudinal study

**DOI:** 10.1177/14034948241236830

**Published:** 2024-03-22

**Authors:** Rose-Marie Johansson-Pajala, Moudud Alam, Annelie K. Gusdal, Lena Marmstål Hammar, Anne-Marie Boström

**Affiliations:** 1 School of Health, Care and Social Welfare, Mälardalen University, Sweden; 2 School of Information and Engineering/Statistics, Dalarna University, Falun, Sweden; 3 School of Health and Welfare, Dalarna University, Falun, Sweden; 4 Division of Nursing, Department of Neurobiology, Care Science and Society, Karolinska Institute, Stockholm, Sweden; 5 R&D unit, Stockholms Sjukhem, Stockholm, Sweden; 6 Theme Inflammation and Aging, Karolinska University Hospital, Huddinge, Sweden

**Keywords:** Home care service, national survey, older adults, register study, safety, security

## Abstract

**Aim:**

Older adults are increasingly encouraged to continue living in their own homes with support from home care services. However, few studies have focused on older adults’ safety in home care. This study explored associations between the sense of security and factors related to demographic characteristics and home care services.

**Methods:**

The mixed longitudinal design was based on a retrospective national survey. The study population consisted of individuals in Sweden (aged 65+ years) granted home care services at any time between 2016 and 2020 (*n*=82,834−94,714). Multiple ordinal logistic regression models were fitted using the generalised estimation equation method to assess the strength of relationship between the dependent (sense of security) and independent (demographics, health and care-related factors) variables.

**Results:**

The sense of security tended to increase between 2016 and 2020, and was significantly associated with being a woman, living outside big cities, being granted more home care services hours or being diagnosed/treated for depression (cumulative odds ratio 2–9% higher). Anxiety, poor health and living alone were most strongly associated with insecurity (cumulative odds ratio 17–64% lower). Aside from overall satisfaction with home care services, accessibility and confidence in staff influenced the sense of security most.

**Conclusions:**

**We stress the need to promote older adults’ sense of security for safe ageing in place, as mandated by Swedish law. Home care services profoundly influence older adults’ sense of security. Therefore, it is vital to prioritise continuity in care, establish trust and build relationships with older adults. Given the increasing shortage of staff, integrating complementary measures, such as welfare technologies, is crucial to promoting this sense of security.**

## Background

Population ageing, which has been associated with a growing number of people experiencing complex health and social needs, has placed increasing demands on the healthcare system [1]. To cope with the challenges of an ageing population, older adults are increasingly encouraged to continue living in their own homes, if needed, with the support of home care services (HCSs) [2, 3]. When living at home, it is important that older adults feel safe and secure with the care provided [4]. Safety is a multidimensional concept which is also referred to as a sense of security. The concepts ‘sense of security’ and ‘safety’ are thus closely related [5]. In home care, sense of security is described as a fundamental and multifaceted concept, encompassing physical, mental, social and practical dimensions [6, 7]. In this study, sense of security refers to an intrinsic state based on confidence and trust in oneself and in others (Swedish: trygghet) [5], rather than, for example, specific physical conditions such as risk of falling, or environmental and economic security.

Supporting older adults in remaining in their own homes and communities is favoured by policymakers and health providers to avoid the costly options of institutional care [8]. In addition, older adults in western countries prefer to live in their own homes for as long as possible [9]. The policy of ageing in place is also employed in Sweden, where about 232,000 older adults were granted HCSs in 2021, compared with about 84,000 living in residential care units [10]. Thus, the need for HCSs is growing, providing a wide range of services such as assistance with personal care and social support for older adults with complex care needs [11]. In the present study we specifically analyse the sense of security among older adults with depression because late-life depression is highly prevalent among older adults, affecting around 5.7% worldwide [12], 17% in Scandinavia [13] and between 5% and 15% in Sweden [14]. These figures apply to older adults in general, while the prevalence of depression among older adults receiving interventions from elder care services is significantly higher [14]. Moreover, late-life depression rarely occurs in isolation and most commonly in conjunction with anxiety disorder [15].

Despite the need to support older adults’ safe living in their own homes [16], few studies have focused on sense of security in home care [6, 17]. Further research is motivated because older adults living in their own homes have been found to be less secure than those living in residential care units [18]. Knowledge on factors affecting older adults’ sense of security at home is also highly motivated by the ongoing transfer in Swedish healthcare to so-called ‘good quality, local healthcare’. The intention is to make primary care instead of hospitals the focal point of care. Hence, more people will receive health and medical care at home [19], including older adults with complex care needs [11]. Furthermore, the growing shortage of home care staff is predicted to become a dire issue in the years ahead [20]. Thus, additional knowledge is needed to guide forthcoming policies and priorities regarding how to provide good-quality HCSs so that older adults can live independently under safe conditions, as stipulated by Swedish law [21]. This study aimed to examine the associations between a sense of security and demographic characteristics and factors related to HCSs.

## Methods

### Design

A retrospective mixed longitudinal study design [22] in which individuals could drop out and new individuals could be included each year of the survey was adopted by relying on the Annual Healthcare Quality Evaluation Survey conducted by the National Board of Health and Welfare (NBHW).

### Study population

The study population consisted of all individuals aged 65 years or over living in Sweden who were granted HCSs any time between 2016 and 2020. As part of their annual quality evaluation of HCSs, NBHW conducts a survey every year around April or May. The questionnaire was sent to every HCSs allottee by ordinary mail and e-mail.

### Outcomes

The questionnaire contained between 24 and 26 items concerning health, contacts with the municipality, influence on care, how the help/service is provided, interactions with staff, sense of security, social activities, accessibility to staff, assistive product usage, and general questions about overall satisfaction with HCSs. If a respondent was unable to answer the survey questions, a relative or staff member, with direct instruction from the respondent, could answer the questionnaire. In this study, a selected subset of items (see Table I) from the survey was utilised. The municipalities were classified into three categories in accordance with the classification scheme adopted by Statistics Sweden (see Table I).

**Table I.** Description of the study variables.

Variable	Question/description	Response alternative/measurement unit	Source
Demographic characteristics			
Year	Survey year	2016, 2017, 2018, 2019, 2020	NBHW’s survey
Municipality	Home care service-procuring municipality	A: Large cities and commuting municipalities near large cities B: Medium-sized towns and commuting municipalities near medium-sized towns C: Other small towns or rural municipalities	NBHW’s survey, SCB (classification)
Age	Age of the respondent	In full calendar years	Patient register
Sex	Sex of the respondent	Male and female	Patient register
Living situation	Do you live together with another adult?	Yes = 1, No = 0	NBHW’s survey
Self-response	Who filled in the survey questionnaire?	1 = I, the care receiver 0 = Proxy respondent (staff, family, relative, etc.)	NBHW’s survey
Self-rated health	What is your assessment of your overall health?	1 = Very good, . . ., 5 = Very bad	NBHW’s survey
Depression	ATC code: N06A, and N06AB, Diagnosis code: ‘F33-’, ‘F33’, ‘F330’, ‘F331’, ‘F332’, ‘F333’, ‘F334’, ‘F338’ and ‘F339’	1 = Depression, 0 = No depression	Drugs and patient register, NBHW
Anxiety	Do you experience distress from uneasiness/worry/anxiety?	1 = No, 2 = Yes, slightly, 3 = Yes, badly	NBHW’s survey
Service granted	HCSs hours consumed within one calendar year	Hours of service consumed per year	SOL data base, NBHW
Alarm	Whether security alarm is installed in the house	1 = Yes, 2 = No	SOL data base, NBHW
Home care services			
Sense of security	How secure or insecure does it feel to live at home with support from home care services?	1 = Very secure, . . ., 5 = Very insecure	NBHW’s survey
Influence	Does the staff consider your opinions and wishes on how the help should be carried out?	1 = Yes, always, . . ., 5 = No, never	NBHW’s survey
Performance of help/services	How do you think the staff members perform their tasks?	1 = Very good, . . ., 5 = Very badly	NBHW’s survey
Punctuality	Does the staff usually arrive at the agreed time?	1 = Yes, always, . . ., 5 = No, never	NBHW’s survey
Sufficient time	Does the staff usually have sufficient time to perform their work at your home?	1 = Yes, always, . . ., 5 = No, never	NBHW’s survey
Treatment	Does the staff usually treat you well?	1 = Yes, always, . . ., 5 = No, never	NBHW’s survey
Respect	Have you experienced any of the following nine incidences^a^ indicating disrespect in your interactions with the staff over the past year?	1 = No, 2 = Yes	NBHW’s survey
Reliability	Do you rely on the staff who come to your home?	1 = Yes, for all staff, . . ., 4 = No, not for anyone on the staff	NBHW’s survey
Accessibility	How easy or difficult is it to get in touch with the home care staff when needed?	1 = Very easy, . . ., 5 = Very difficult	NBHW’s survey
Overall satisfaction	Overall, how satisfied or dissatisfied are you with the home care service you have received?	1 = Very satisfied, . . ., 5 = Very dissatisfied	NBHW’s survey

a Respect for privacy, negative comments, treated disrespectfully, treated like a child, denied wishes for help, denied wishes at mealtimes, disrespect in toileting, bathing and dressing and keeping distance in nursing.

In addition to the survey, data on the diagnosis and medication for the treatment of depression, birth year and sex were extracted from the patient and medication register maintained by NBHW (see Table I). A respondent was considered to have depression if, between January and May of the same survey year, the individual was prescribed medication with ATC code ‘N06A’ (antidepressants) or was diagnosed with the following diagnosis codes: ‘F33-’, ‘F33’, ‘F330’, ‘F331’, ‘F332’, ‘F333’, ‘F334’, ‘F338’ or ‘F339’ (recurrent depressive disorder). Data on actual hours of granted service and the use of safety alarms were also extracted from another register (SOL) maintained by NBHW.

### Statistical analysis

Descriptive statistics were used to summarise the study variables, assess the representativeness of the sample and determine the plausibility of the assumption about the missingness mechanism [23]. The survey non-response mechanism was assumed to be missing completely at random (MCAR) [23]. Under MCAR, we treated the survey respondents as a random sample of the population. Item non-response was also treated as MCAR and deleted list-wise in the analysis. The ordinal logistic (or proportional odds (PO)) regression model was fitted to infer the factors affecting the sense of security. All data preparation and analysis were conducted using R statistical software 2023. PO models were fitted using the generalised estimation equation (GEE) technique by using the ordLORgee function from the multgee package in R.

### Ethics

Informed consent was obtained from the respondents by NBHW. Data were coded, and no respondent could be identified in the study. The respondents were informed by NBHW that their data could be used in research. The study was approved by the Regional Research Ethics Committee of Uppsala, Sweden (reg. no. 2017/140). Ethical standards for scientific work were followed [24].

## Results

The frequency of item non-response was very low, with a maximum of (approximately) 13% of responses missing for the accessibility question. Between 7% (in 2020) and 13% (in 2016) of responses for proxy status were also missing. For the sense of security question, 8% of responses were missing. Most of the variables had a non-response rate below 5%. However, there was at least one missing value for any of the study variables in 40.3% of all incidences.

Summary statistics of the study variables are presented in Table II. Overall, the study population’s characteristics and response patterns were very similar over the years. The study population consisted predominantly of older individuals (at least 75% of the respondents were aged 79 years or over), two-thirds were women (65–68%) and a majority lived alone. Between 28% and 30% of the respondents received medication for or were diagnosed with depression each survey year (January–May). Most reported having good to average self-rated health; only around 22–24% had poor to very poor health. Most responded that they were treated well by the staff. However, a noticeable proportion (18%) mentioned that the staff did not have sufficient time to perform their work and that it was not easy to contact them (22%). Interactions with staff involving negative incidences, indicating disrespect, had also increased slightly over the years since 2016.

**Table II.** Summary statistics of the study variables.

Variable	Year				
	2016	2017	2018	2019	2020
Demographic characteristics					
Number of respondents	94,714	91,634	87,390	88,749	82,834
Age, median (Q1, Q3)	86 (80, 90)	86 (80, 90)	86 (80, 90)	86 (79, 90)	86 (79, 90)
Sex, female	67%	68%	68%	66%	65%
Living situation, living alone	76%	76%	75%	75%	74%
Self-response, proxy response	43%	42%	40%	38%	31%
Self-rated health, somewhat to very bad	24%	24%	24%	23%	22%
Depression (count)	28% (26,926)	28% (26,018)	29% (25,151)	29% (26,160)	30% (24,704)
Anxiety, slightly to bad	49%	49%	49%	49%	47%
Mean hours of service granted (SD)	376.2 (409.8)	363.9 (406.0)	365.8 (408.2)	370.3 (403.3)	354.2 (390.1)
Alarm, with security alarm	66%	66%	66%	71%	72%
Home care services					
Sense of security, neither secure nor insecure to very insecure					
With depression	16%	15%	16%	15%	16%
Without depression	14%	14%	14%	14%	14%
Overall	15%	14%	15%	14%	14%
Influence on provided care, sometimes to never	13%	13%	14%	14%	13%
Performance of help/services, average to very poorly	13%	13%	14%	14%	14%
Punctuality, sometimes to never	14%	15%	16%	16%	16%
Sufficient time, sometimes to never	18%	18%	18%	18%	18%
Treatment, sometimes to never	3%	3%	3%	3%	3%
Respect, experienced bad incidences indicating disrespect	14%	14%	15%	15%	16%
Reliability, no confidence in most of or any staff	9%	9%	10%	10%	11%
Accessibility, neither easy nor difficult to very difficult	22%	21%	23%	23%	23%
Overall satisfaction, neither satisfied or not dissatisfied to very dissatisfied	11%	11%	12%	12%	12%

PO logistic models were fitted using the GEE approach to the subsets of data pertaining to persons with depression and without depression separately, as well as to the full data set consisting of both groups. The response variable was the self-rated feeling of security, which was rated on a five-point Likert scale (very secure being the lowest category and very insecure being the highest category). The results from the PO model are presented in Table III. Any cumulative odds ratio (COR) greater than 1 indicates a positive association with the state of security – that is, increase in the respective independent variable (or compared with the baseline category) leads to higher cumulative odds of a response towards secure to more secure than not secure.

**Table III.** Cumulative odds ratio estimate from the PO models of sense of security (response order: very secure < quite secure < neither secure nor insecure < quite insecure < very insecure).

Effect	All			Depression			No depression		
	COR	95% CI		COR	95% CI		COR	95% CI	
Demographic characteristics									
Year; 2017 (ref. year 2016)	1.04	1.01	1.06	1.04^n^	1.00	1.09	1.04	1.01	1.07
2018	1.07	1.04	1.10	1.07	1.03	1.12	1.07	1.04	1.10
2019	1.11	1.09	1.14	1.13	1.08	1.18	1.11	1.08	1.14
2020	1.14	1.11	1.17	1.12	1.07	1.17	1.16	1.12	1.19
Mun. type; B (ref. A)	1.06	1.04	1.09	1.06	1.02	1.10	1.07	1.04	1.09
C	1.03	1.01	1.06	1.05	1.00	1.09	1.03^n^	1.00	1.06
Sex; female (ref. male)	1.02	1.00	1.05	0.99^n^	0.96	1.03	1.03	1.01	1.06
Living alone (ref. live with others)	0.83	0.81	0.85	0.85	0.81	0.88	0.83	0.80	0.85
Proxy response (ref. self)	0.98^n^	0.97	1.00	0.91	0.88	0.94	1.02	1.00	1.05
Self-rated health; bad (ref. good)	0.66	0.64	0.67	0.65	0.63	0.68	0.65	0.64	0.67
Depression (ref. no depression)	1.07	1.05	1.09						
Anxiety; slightly (ref. no anxiety)	0.58	0.57	0.59	0.59	0.57	0.62	0.57	0.56	0.59
Badly	0.36	0.35	0.37	0.37	0.35	0.39	0.35	0.34	0.37
Log. (1 + granted service time)	1.09	1.08	1.10	1.09	1.08	1.10	1.09	1.08	1.10
Security alarm; yes (ref. no)	0.97	0.95	0.99	0.96	0.93	1.00	0.97	0.95	0.99
Home care services									
Influence; often (ref. always)	0.78	0.76	0.79	0.77	0.74	0.80	0.78	0.76	0.80
Sometimes	0.74	0.71	0.77	0.76	0.71	0.80	0.73	0.70	0.77
Hardly any	0.67	0.63	0.71	0.69	0.63	0.77	0.67	0.62	0.72
Never	0.70	0.63	0.77	0.58	0.49	0.69	0.76	0.68	0.86
Help/service; good (ref. very good)	0.73	0.72	0.75	0.73	0.70	0.76	0.73	0.71	0.76
Neither well nor bad	0.61	0.59	0.64	0.64	0.60	0.68	0.60	0.57	0.63
Bad	0.60	0.56	0.65	0.60	0.54	0.67	0.60	0.55	0.66
Very bad	0.44	0.39	0.50	0.43	0.35	0.53	0.45	0.39	0.53
Punctuality; often (ref. always)	0.76	0.75	0.78	0.77	0.74	0.81	0.76	0.74	0.78
Sometimes	0.74	0.72	0.77	0.77	0.72	0.82	0.73	0.70	0.77
Seldom	0.74	0.70	0.78	0.74	0.68	0.81	0.74	0.70	0.78
Never	0.75	0.69	0.81	0.78	0.67	0.90	0.74	0.67	0.81
Suff. time; often (ref. always)	0.77	0.76	0.79	0.75	0.72	0.78	0.78	0.76	0.80
Sometimes	0.71	0.69	0.74	0.71	0.67	0.75	0.72	0.69	0.75
Seldom	0.66	0.63	0.68	0.66	0.61	0.71	0.65	0.62	0.69
Never	0.62	0.58	0.66	0.61	0.54	0.68	0.62	0.57	0.67
Good treatment; often (ref. always)	0.87	0.85	0.89	0.90	0.86	0.94	0.86	0.84	0.88
Sometimes to never	0.71	0.67	0.76	0.72	0.65	0.79	0.72	0.67	0.77
Respect; exp. bad incident (ref. no)	1.04	1.01	1.07	1.06	1.01	1.10	1.03	1.00	1.07
Reliability; conf. in many (ref. all)	0.66	0.64	0.67	0.66	0.64	0.69	0.65	0.64	0.67
In a few staff	0.41	0.40	0.43	0.41	0.38	0.44	0.41	0.40	0.43
No confidence in any	0.30	0.26	0.34	0.26	0.21	0.33	0.32	0.27	0.38
Accessibility; easy (ref. very easy)	0.53	0.51	0.54	0.53	0.51	0.55	0.52	0.51	0.53
Neither easy nor difficult	0.40	0.39	0.41	0.39	0.37	0.41	0.40	0.39	0.42
Quite difficult	0.38	0.37	0.40	0.38	0.35	0.40	0.39	0.37	0.40
Very difficult	0.34	0.32	0.36	0.32	0.28	0.36	0.35	0.32	0.38
Overall. satis.; satisfied	0.41	0.40	0.42	0.40	0.38	0.42	0.41	0.40	0.43
Neutral	0.17	0.16	0.18	0.17	0.15	0.18	0.17	0.16	0.18
Quite dissatisfied	0.10	0.09	0.11	0.10	0.09	0.11	0.10	0.09	0.10
Very dissatisfied	0.03	0.03	0.04	0.03	0.03	0.04	0.03	0.03	0.04

n Not significant at 5% level; all other effects are significant.

Cumulative odds ratio (COR) >1 indicates higher odds to answer towards secure than insecure.

CI: confidence interval; PO: proportional odds.

The direction of association between the independent and dependent variables was found to be the same across the three separate analyses (Table III), although the exact magnitudes of the effects were slightly different (such differences are mostly ignorable). Most of the study variables were negatively associated with a sense of security. However, women, people with depression and those who lived outside the big cities and were granted more service hours felt slightly more secure (change in COR <10%, for most cases). The sense of security tended to get better over the years (in that all year effects were positive, and the estimates increased in magnitude over the years; Table III). Aside from overall satisfaction with HCSs, accessibility and confidence in the staff were the most influential variables (in terms of the magnitude of the COR) for the sense of security. Those who rate the accessibility to the staff as very difficult or not having confidence in any of the staff had about 62–79% lower cumulative odds of feeling more secure than insecure. Those who were very dissatisfied with the overall service quality of HCSs also had 97% lower odds of feeling secure than those who were very satisfied with the overall service quality.

## Discussion

In this nationwide register study, we found that the sense of security among older adults living in their own homes with HCSs tended to increase from 2016 to 2020. This result can be considered somewhat surprising in view of the ongoing discussions in society about the growing demands on welfare services and the increasing shortage of home care staff [1, 20]. However, approximately 10,800 to 12,800 (14–16%) older adults do not feel entirely secure living in their own homes with support from HCSs. Swedish law stipulates that older adults should be able to live independently under safe conditions [21]. Therefore, additional measures are needed to enable safe ageing in one’s own home, specifically considering the ongoing transfer to ‘good quality, local healthcare’, which means that more people will receive health and medical care at home instead of in hospitals [19].

### Associations between a sense of security and demographic characteristics

The findings suggest that being a woman, living outside big cities and receiving more HCSs hours were associated with an increased sense of security (2–9% higher odds of sense of security). On the contrary, anxiety, poor health and living alone were most strongly associated with insecurity (17–64% lower odds for sense of security). These findings correspond with previous research showing that living alone and poor health are significantly associated with insecurity [17, 25]. An interesting finding is the higher odds of a sense of security when living outside big cities. This could perhaps be related to organisational or staffing issues; however, further research is needed.

The findings also showed that older adults diagnosed with/treated for depression had 7% higher odds of a sense of security compared with the no depression group. The incidence of depression and mental disorders is known to be higher in older adults who have interventions from care services than in those who do not. Moreover, suicide is more common among older adults experiencing mental disorders than among younger people [14]. However, our results suggest that regarding a sense of security, which can be considered an important component of general wellbeing, older adults diagnosed with/treated for depression were not more insecure than those who were not. On the contrary, adults with depression felt more secure. It was not possible to draw any extensive conclusions from these slightly increased odds of a sense of security among those diagnosed/treated for depression; a possible explanation could stem from care in general and medication.

The results revealed that the proportion of older adults who were diagnosed with/treated for depression increased from 28% to 30% between 2016 and 2020. These figures can be considered high compared with those reported by NBHW (5–15%). However, the prevalence of depression and mental disorders is significantly higher among older adults with interventions from care services including HCSs [14]. Notably, almost half of the respondents experienced anxiety, which often co-occurs with depression [15], but still, most respondents reported average to good health.

### Associations between a sense of security and factors related to HCSs

The results show that eight of the nine study variables related to HCSs were negatively associated with a sense of security, which implies that older adults’ sense of security is highly affected by quality of service. The factors most strongly associated with a sense of security were the overall satisfaction with their HCSs, the accessibility to the staff (i.e. to get in touch with the staff when needed) and reliability (i.e. if they rely on the staff who come to their home). As many as 18% of the older adults stated that the staff did not have sufficient time to do their jobs, and 22% stated that it was not easy to contact the staff. Those who did not have confidence in the staff had significantly lower odds of experiencing a sense of security. The associations between a sense of security and HCSs did not differ significantly between older adults diagnosed with/treated for depression and those without depression, except regarding the influence on how care should be carried out. The findings confirm previous research showing that older adults need to feel that they can trust the staff to feel safe. Other factors, such as family, environment and the older adults themselves, also have an impact on the sense of security [6]. However, a good relationship with home care staff is a widely emphasised condition for a sense of security [4, 6, 18]. One may question how home care staff might build relationships and trust with older adults in Sweden when they meet, on average, 16 different home care staff every fortnight [26]. We cannot comment on continuity in the present study, as this was not investigated; however, continuity in staff is a key factor in nurturing relationships between home care staff and older adults [27]. These relationships are a prerequisite for person-centred care, which in turn is a cornerstone of the ongoing transfer to ‘good quality, local healthcare’ [19].

A prerequisite for staff to be able to support older adults adequately is that they themselves experience a sense of security [5]. Older adults’ sense of security has been found to be strongly associated with the staff’s working environment, including psychological working conditions and job strain factors. Therefore, managers and policymakers need to acknowledge that the working conditions of home care staff are important for older adults’ satisfaction with HCSs [2]. In addition, staff must have sufficient knowledge and competence to provide good and safe care [11]. The requirements for adequate education and skills can conceivably become more rigorous, considering that the staff will need to care for an increasing number of severely ill older adults. At the same time, the staff shortage is becoming increasingly acute [20]. The reduction in welfare resources means that technical solutions will be given more emphasis to generating a sense of security [5]. Increasing the sense of security with the use of welfare technology is also a clearly stated goal of policy documents [29]. The present study provides no support that safety alarms increase a sense of security, but new welfare technologies are developing at a rapid pace. Digital security alarms, night surveillance cameras, communication technology and GPS alarms are examples of welfare technology that can promote a sense of security in the home [28]. However, research is still limited regarding the extent to which this type of technology contributes to a sense of security. Thus, more research is needed to evaluate the effects [29].

### Strengths and limitations

A major strength of this study is that it is longitudinal and based on a nationwide survey with a large sample. In addition, the frequency of item non-response was very low. One limitation is that the sense of security can be defined and measured in different ways. Thus, the single-question item on the sense of security in the questionnaire may not account for various dimensions of the concept. The same applies for anxiety, which also is a concept with several dimensions. However, the study was based on an annual survey conducted by NBHW that did not include any additional measurements.

The results obtained from the statistical model may be sensitive to the covariates included in the model. Such a critique is applicable to any statistical model, especially when the independent variables are correlated. Some of the results from the statistical model (Table III) were not explainable (e.g. someone who experienced an incidence of disrespect but also felt more secure). Such contradictory results might be a consequence of multicollinearity or omitted variable bias, but we did not dig further into this issue.

The occurrence of depression among the respondents was based on diagnosis and medication records between January and May of each survey year, which may be considered a limitation, as these diagnoses may have been underreported. Moreover, antidepressants can be prescribed for reasons other than depression.

## Conclusions and clinical implications

To conclude, the overall sense of security tended to increase between 2016 and 2020, but 14–16% of older adults reported not feeling entirely secure with support from HCSs. Being a woman, living outside big cities, being granted more HCSs hours or being diagnosed/treated for depression were associated with an increased sense of security, while anxiety, poor health and living alone were most strongly associated with insecurity. Overall satisfaction with HCSs was significantly associated with a sense of security, as was accessibility and confidence with the staff.

We stress the need to promote older adults’ sense of security for safe ageing in place, as mandated by Swedish law. HCSs profoundly influence their sense of security. Therefore, it is vital to improve the staffs’ prerequisites to ensure good and safe care by prioritising continuity, establishing trust and building strong relationships with older adults. This would also benefit the healthcare system, as increasingly more healthcare services will be carried out in peoples’ homes. Staff can gain a deeper understanding of the person, allowing them to detect any declines in health more promptly. Given the increasing shortage of home care staff, more research is needed on complementary solutions, such as welfare technologies and their effects on older adults’ sense of security. Notably, nearly half of the respondents experienced anxiety, underscoring the need for continued research in this area.
